# Imaging Manifestations of Mediastinal Fat Necrosis

**DOI:** 10.1155/2013/323579

**Published:** 2013-11-28

**Authors:** Malay Y. Bhatt, Santiago Martínez-Jiménez, Melissa L. Rosado-de-Christenson, Kenneth R. Watson, Christopher M. Walker, Jeffrey R. Kunin

**Affiliations:** ^1^University of Missouri-Kansas City School of Medicine, 2411 Holmes Street, Kansas City, MO 64108, USA; ^2^The Department of Radiology, Saint Luke's Hospital of Kansas City, 4401 Wornall Road, Kansas City, MO 64111, USA; ^3^The Department of Pathology, Saint Luke's Hospital of Kansas City, 4401 Wornall Road, Kansas City, MO 64111, USA

## Abstract

Mediastinal fat necrosis (MFN) or epipericardial fat necrosis, as it is commonly referred to in the literature, is a rare self-limiting cause of chest pain of unclear etiology. MFN affects previously healthy individuals who present with acute pleuritic chest pain. Characteristic computed tomography (CT) findings include a fat attenuation lesion with intrinsic and surrounding increased attenuation stranding. There is often associated thickening of the adjacent pericardium and/or pleural effusions. We present two cases of MFN manifesting as ovoid fat attenuation lesions demarcated by a soft tissue attenuation rim with intrinsic and surrounding soft tissue attenuation stranding and review the clinical and pathologic features of these lesions. Knowledge of the clinical presentation of patients with MFN and familiarity with the characteristic imaging findings of these lesions should allow radiologists to prospectively establish the correct diagnosis and suggest conservative management and follow-up.

## 1. Introduction

Mediastinal fat necrosis (MFN) is a rare self-limiting cause of chest pain, with the first reported cases dating back to 1957 [1]. In the current literature, “epipericardial” or “epicardial” fat necrosis is the term used to identify this condition [2–4]. However, as the juxtapericardial mediastinal fat is characteristically affected, we propose that the term MFN is more appropriate given the anatomical location of the disease process. MFN classically affects previously healthy individuals who present with acute pleuritic chest pain that raises concern for an acute cardiopulmonary process including pulmonary thromboembolic and coronary artery diseases [1–12]. We present two cases of MFN and discuss their clinical, pathologic, and imaging findings. In both cases, the affected patients presented with severe chest pain and no associated physical examination findings or specific laboratory abnormalities. The presentation of acute pleuritic chest pain in association with CT findings of an ovoid juxtapericardial fat attenuation lesion with intrinsic and surrounding increased attenuation stranding, thickening of the adjacent pericardium, and resolution on follow-up imaging can be collectively used to establish the diagnosis [2]. Making the correct diagnosis prospectively mitigates unnecessary testing in favor of conservative management.

## 2. Case Reports

**Case 1**

**Figure 1:**
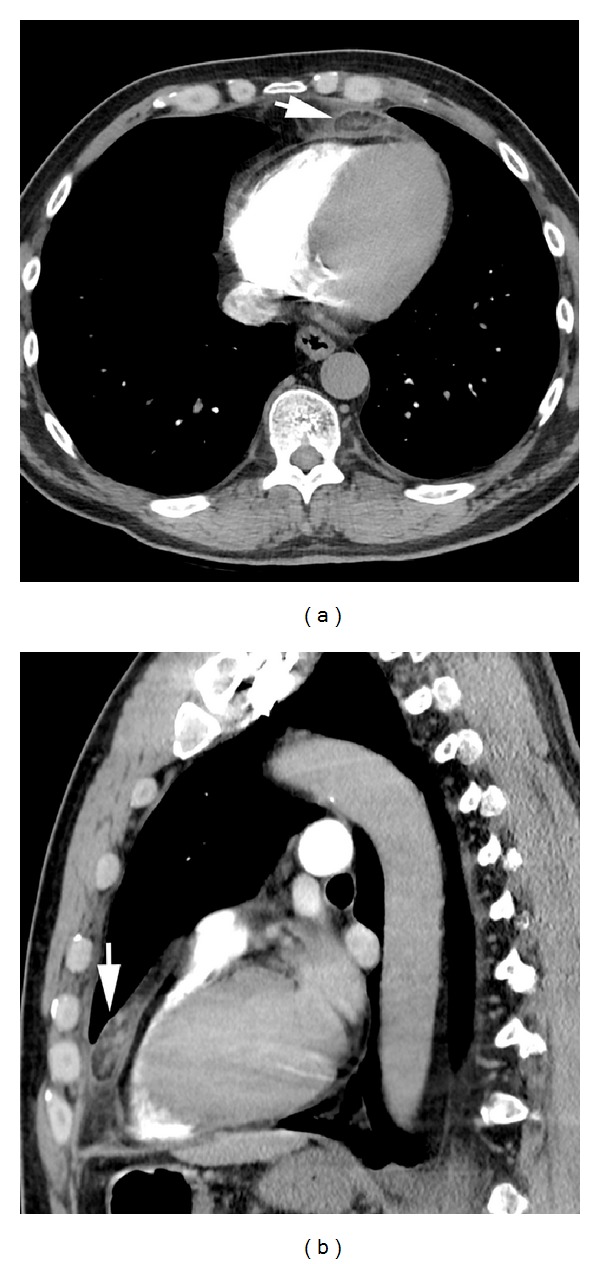
Axial (a) and sagittal (b) contrast-enhanced chest CT (soft tissue window) shows an anterior mediastinal ovoid fat attenuation lesion (arrows) with intrinsic and surrounding soft tissue attenuation stranding. The lesion is demarcated by a thin rim of soft tissue attenuation and abuts the adjacent pericardium. Note small left pleural effusion (b).

A 51-year-old man presented with dyspnea and left pleuritic chest pain that radiated to his back. His physical exam and laboratory tests were normal. PA and lateral chest radiographs showed a small left pleural effusion. Chest CT showed a 3.2 × 1.3 × 3.2 cm (transverse, AP, and crainocaudad, resp.) ovoid fat attenuation lesion delimited by a thin soft tissue attenuation rim in the juxtapericardial anterior mediastinal fat (Figure 1). The lesion exhibited intrinsic soft tissue stranding and surrounding soft tissue attenuation that abutted the adjacent pericardium. The patient was discharged on nonsteroidal anti-inflammatory drugs and analgesics. Follow-up CT was recommended but was not performed. An echocardiogram which was performed one year later showed no abnormality.

**Case 2**

**Figure 2:**
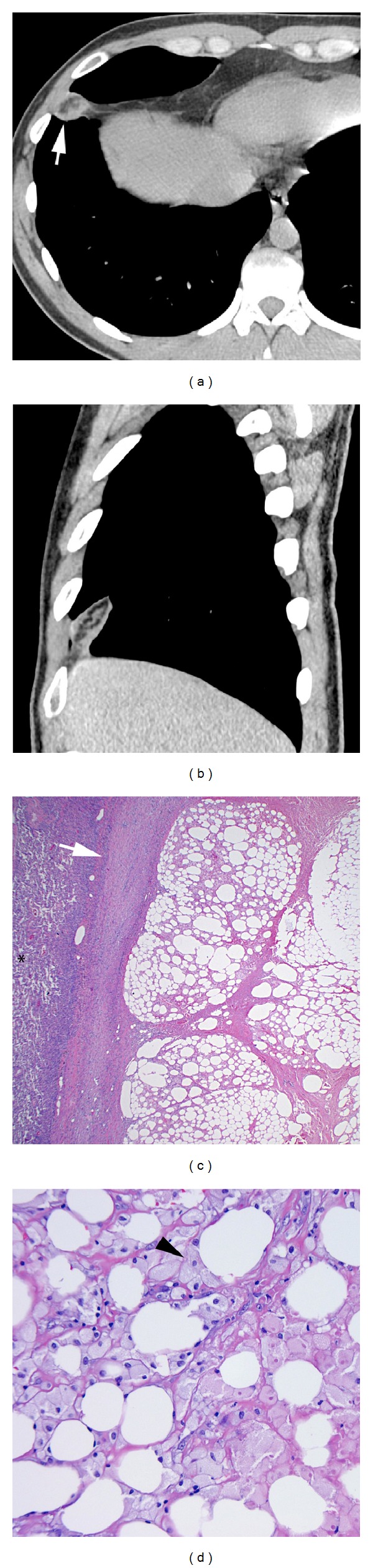
Axial (a) and oblique (b) unenhanced chest CT (soft tissue window) shows an ovoid lesion (arrow) of heterogeneous fat attenuation with intrinsic soft tissue stranding in the inferior aspect of the right major fissure. The lesion is surrounded by a thick rim of soft tissue attenuation. (c) Low power photomicrograph (hematoxylin & eosin [H&E] stain; original magnification 40x) shows lobules of adipose tissue surrounded by a fibrous pseudocapsule (arrow) and adjacent atelectatic lung parenchyma (*). (d) High power photomicrograph (H&E stain; 400x) shows fat necrosis and lipid laden macrophages (arrowhead).

A 30-year-old man presented with a four-day history of right pleuritic chest pain that was sharp, originated in the right hemithorax, and radiated to the back. He was afebrile, had a normal physical examination, and had no positive laboratory findings other than an elevated C-reactive protein. PA and lateral chest radiographs showed a small right pleural effusion and no consolidation. Chest CT showed a 3.1 × 1.9 × 3.7 cm (transverse, AP, and craniocaudad, resp.) ovoid fat attenuation lesion with intrinsic soft tissue stranding within the inferior aspect of the right major fissure (Figures 2(a) and 2(b)). The lesion was demarcated by a soft tissue attenuation rim. There was an associated small ipsilateral pleural effusion. The patient was initially treated with intravenous antibiotics without resolution of symptoms. Follow-up chest CT showed resolution of the right pleural effusion but no change in the lesion within the inferior right major fissure. Given persistence of symptoms and clinical concerns for a pleural neoplasm, the patient underwent video-assisted thoracoscopic resection 19 days after his initial presentation. On gross examination, the lesion appeared to be fatty and well-circumscribed and exhibited evidence of acute hemorrhage. The pleura overlying the lesion exhibited fibrosis and fibrinous adhesions. Frozen section revealed fat necrosis. Permanent sections showed lobules of necrotic adipose tissue with a surrounding fibrous pseudocapsule (Figures 2(c) and 2(d)). The patient had a favorable postoperative course and remained clinically stable with improvement and eventual resolution of his pain.

## 3. Discussion

MFN is a rare self-limiting cause of acute chest pain of unclear etiology. Since the initial description of this condition in 1957, the designation of pericardial fat necrosis was used [1]. Recent reports disputed this nomenclature, stating that pericardial fat necrosis is a misnomer [2, 3]. As MFN characteristically occurs within the mediastinum outside the pericardium, the term epipericardial fat necrosis was proposed and adopted [2, 3]. However, as the lesion usually occurs in the juxtapericardial mediastinal fat, MFN seems a more appropriate term to designate this lesion. In addition, it should be noted that mediastinal fat may extend into the adjacent interlobar fissures, explaining the atypical findings and location of MFN described in Case 2 [4, 13].

Patients with MFN characteristically present with acute pleuritic chest pain that may mimic other acute cardiopulmonary processes, including myocardial infarction, pulmonary embolism, pneumonia, and acute pericarditis [5, 9]. The history of trauma or infection is usually absent. Chest pain is usually ipsilateral to the lesion, which is more commonly on the left but can be right-sided [4]. The pain is often intermittent and worsens with movement and deep inspiration [3]. Syncope, dyspnea, and tachycardia have also been reported [4, 8]. A pericardial friction rub has been described on auscultation [3, 4]. Although there are usually no abnormal laboratory findings, our second patient had nonspecific elevation of C-reactive protein. Electrocardiography is normal in most cases, although nonspecific ST-T changes, right bundle branch block, paroxysmal atrial tachycardia, and a prior myocardial infarction have been reported in four cases [4].

Since its initial description, the diagnosis of MFN was based on pathologic examination of resected tissue [1, 3]. In fact, twenty-one of the previously reported twenty-six cases were treated surgically [4]. With the increased utilization of CT, specific findings of MFN have been described. When correlated with the clinical presentation, these findings have been sufficient to make the diagnosis, and affected patients have avoided surgery [2]. CT allows localization of the lesion in the juxtapericardial mediastinal fat [7, 11].

Early in the disease course, chest radiographs may be normal, although a juxtacardiac opacity near the cardiophrenic angle with or without associated pleural effusion may be seen [9]. This abnormality is usually ipsilateral to the chest pain and is contiguous with the cardiac silhouette [3]. Both of our patients had pleural effusions ipsilateral to their chest pain on radiography, and in one of our cases the lesion was visible as a triangular intrafissural opacity (indistinguishable from intrafissural mediastinal fat) on a lateral chest radiograph. Chest CT allows localization and characterization of the lesion and facilitates the prospective diagnosis of MFN. The most common finding is an “encapsulated” fat attenuation lesion with intrinsic increased attenuation stranding [2–9]. There is often soft tissue stranding of the surrounding mediastinal fat [2–9]. There may also be thickening of the adjacent pericardium and pleural effusions [2–9] (Figure 1(b)). Both of our patients had fat attenuation lesions demarcated by a soft tissue attenuation rim that resembled a capsule. The lesions exhibited intrinsic and surrounding soft tissue attenuation stranding. Our first patient exhibited typical findings in a characteristic location (Figure 1). Our second patient showed typical findings of MFN within intrafissural mediastinal fat, an atypical location [4] (Figures 2(a) and 2(b)). However, a recent review of this unusual entity, in which the lesion is designated as “epicardial fat necrosis,” describes 5 similar lesions that may have extended into the pleural fissures [1, 4].

The CT finding of an ovoid mediastinal fatty lesion with a soft tissue rim and intrinsic and surrounding soft tissue stranding is important not only in making the diagnosis of MFN but also in differentiating it from other fat containing mediastinal lesions [11, 14]. These lesions include Morgagni hernia, lipoma, liposarcoma, and thymolipoma [11, 14]. Morgagni hernia manifests with migration of omental fat (and sometimes other abdominal contents) through a defect in the right anteromedial hemidiaphragm [11]. Lipoma, like MFN, manifests as a fat density mass but is usually asymptomatic, has no surrounding or intrinsic soft tissue stranding or pericardial thickening, and does not resolve on follow-up imaging [3, 14]. While liposarcoma may exhibit fat attenuation, it is often a large locally invasive mass with significant soft tissue attenuation and mass effect on adjacent structures [11]. Thymolipoma manifests as a heterogeneous anterior mediastinal mass arising in the thymus and exhibiting an admixture of fat and soft tissue elements [11, 14].

The etiology of MFN remains unknown, but three theories have been postulated. The first proposes acute torsion of mediastinal fat causing necrosis [10]. The second proposes that necrosis relates to the Valsalva maneuver, which may cause fluctuations in intrathoracic venous pressures that may lead to hemorrhage into the mediastinal fat [2, 9, 10]. Lastly, an underlying abnormality, such as lipomatosis or lipoma, may predispose to fat necrosis from local trauma related to cardiac and diaphragmatic motion [8, 9, 12].

The pathologic appearance of MFN varies with the duration of symptoms and the age of the lesion [2, 7–9]. Grossly, the lesion is described as a yellow fatty mass with tissue strands extending into the adjacent adipose tissue and a local inflammatory reaction [3, 10]. Microscopically, early lesions reveal fat necrosis with acute inflammatory infiltrates and lipid-laden macrophages [2, 7–9]. Older lesions are often completely surrounded by a fibrous pseudocapsule with fibrous septa surrounding necrotic lobules of adipose tissue, as seen in our second case [2, 7–9] (Figure 2(c)). In this case, the fibrous pseudocapsule was densely adherent to the adjacent thickened and fibrotic pleura. We suggest that this finding correlates with the soft tissue attenuation rim that may surround these lesions on CT studies. Such older lesions also exhibit sheets of lipid-laden macrophages that replace the necrotic fat (Figure 2(d)). There is often an associated foreign body giant cell reaction with giant cells containing fat globules [2, 7–9].

MFN shares many imaging and pathologic features with fat necrosis seen elsewhere in the body [2, 5–9, 11, 15]. For example, epiploic appendagitis is a similar benign self-limiting lesion seen in patients who present with acute abdominal pain [15]. This lesion was previously unknown to radiologists. However, after its description in the imaging literature, epiploic appendagitis is increasingly diagnosed on abdominal CT, and affected patients are successfully managed conservatively [15–17]. A review of the cases of MFN reported in the English literature shows that 21 of 26 cases were treated surgically [4]. However, there are 4 reported cases of MFN with successful conservative management similar to that seen in cases of epiploic appendagitis [2, 4, 11, 15–17]. In fact, the prospective diagnosis of MFN is critical as conservative management is now considered to be the standard of care [2–7, 9]. The triad of acute pleuritic chest pain, CT showing a fatty lesion in the mediastinum with intrinsic and surrounding soft tissue stranding, and thickening of the adjacent pericardium should suggest the diagnosis [2]. We suggest that the identification of a soft tissue attenuation rim surrounding the lesion may be helpful in distinguishing it from other fat containing mediastinal lesions. As our second case shows, MFN may also affect intrafissural mediastinal fat. Resolution or decrease in size of the lesion in serial imaging helps confirm the diagnosis [2–5, 7]. The preferred treatment is nonsteroidal anti-inflammatory drugs [2–5, 7].

In summary, MFN is a rare self-limiting cause of acute pleuritic chest pain. A confident prospective diagnosis based on CT findings may help preclude unnecessary invasive procedures. Acute chest pain with a negative workup for acute coronary syndrome and other cardiopulmonary processes together with characteristic CT findings should suggest the diagnosis. Due to the anatomic location of the actual necrosis and the fact that mediastinal fat may extend into adjacent fissures, we propose that this condition hereon be identified as mediastinal fat necrosis.
